# Recent advances in marburgvirus research

**DOI:** 10.12688/f1000research.17573.1

**Published:** 2019-05-21

**Authors:** Judith Olejnik, Elke Mühlberger, Adam J. Hume

**Affiliations:** 1 Department of Microbiology, Boston University School of Medicine, Boston, Massachusetts, 02118, USA; 2 National Emerging Infectious Diseases Laboratories, Boston University, Boston, Massachusetts, 02118, USA

**Keywords:** Marburg virus, marburgviruses, filovirus, filoviruses, Egyptian rousette, viral proteins

## Abstract

Marburgviruses are closely related to ebolaviruses and cause a devastating disease in humans. In 2012, we published a comprehensive review of the first 45 years of research on marburgviruses and the disease they cause, ranging from molecular biology to ecology. Spurred in part by the deadly Ebola virus outbreak in West Africa in 2013–2016, research on all filoviruses has intensified. Not meant as an introduction to marburgviruses, this article instead provides a synopsis of recent progress in marburgvirus research with a particular focus on molecular biology, advances in animal modeling, and the use of Egyptian fruit bats in infection experiments.

## Introduction

Marburg virus (MARV) is a member of the *Marburgvirus* genus that contains two different viruses: MARV and Ravn virus (RAVV). Both viruses are represented by numerous isolates ^1^. Filovirus taxonomy is confusing, and for those who do not know the difference between Marburg virus (the virus MARV), *Marburgvirus* (the genus), and marburgvirus (MARV and RAVV), we recommend browsing through the *Guide to the Correct Use of Filoviral Nomenclature* by Kuhn ^2^.

MARV is closely related to the better-known Ebola virus (EBOV) and causes a similarly severe disease in humans. Some of the unique characteristics of filovirus outbreaks were reported for MARV disease (MVD) long before they were noticed in EBOV disease (EVD). This includes persistent infection, sexual transmission, and long-term sequelae ^3^. There are also heart-breaking reports of social stigmatization and severe chronic health issues as recalled by survivors of the 1967 MVD outbreak in Marburg, Germany ^4^. Notably, one of the patients from that outbreak temporarily lost the ability to write and calculate and never completely recovered from concentration disorders, reflecting the severe neurological consequences in survivors of filovirus disease ^4^.

MVD remains a global health threat with outbreaks continuing to occur in Central Africa, including two outbreaks in Uganda in 2012 and 2014 ^5, 6^. Jointly with EVD, MVD is listed on the World Health Organization 2018 Priority Diseases List (https://www.who.int/blueprint/priority-diseases/en/). This list is used as a tool to determine which diseases and pathogens should be prioritized for research, development of countermeasures, and emergency response preparedness.

In 2012, we published a comprehensive overview on marburgviruses and the disease they cause, ranging from ecology to molecular biology and treatment options ^7^. A number of areas of significant progress in the marburgvirus field since 2012—including the development of tools to study MARV replication and transcription and to rescue MARV clones ^8, 9^, advances in filovirus countermeasures ^10, 11^, and vaccine development ^12^—have recently been summarized. Progress in the development of MARV vaccines and antiviral treatment options has led to phase 1 clinical trials to evaluate their safe use in humans ^13– 15^. Rather than repeating what has been covered in our previous review and these other excellent reviews, this article instead will focus on (1) recent progress in marburgvirus molecular biology, (2) novel developments in the study of marburgviruses in Egyptian fruit bats, and (3) advances in the use of animal models to study marburgvirus infection, including their use in resolving isolate-specific differences in virulence and pathogenicity.

## 1. Molecular biology

During the last 6 years, much research has focused on a more detailed understanding of the different steps of the marburgvirus replication cycle as well as virus–host interactions using crystal structure, biochemistry, and bioinformatics approaches. Live-cell imaging studies have been instrumental in developing a deeper understanding of marburgvirus assembly and particle release.

### a. Marburg virus genome

Marburgviruses belong to the group of non-segmented negative-strand RNA viruses. A detailed overview of viral genome organization, cis-acting elements, and genome replication and transcription strategies is provided in 7, 16. High-throughput sequencing of MARV RNA combined with bioinformatics and statistical analysis has provided new insights into viral genome plasticity and MARV evolution. This includes codon usage analysis ^17^, phylogenetics ^18, 19^, and the identification of hot spots of U–C substitutions ^20, 21^. Although the function of these U–C substitutions is unknown, it is suggestive of adenosine deaminase (ADAR) editing ^20, 21^. Deep sequencing of MARV Angola RNA obtained from infected cells and infected non-human primates (NHPs) determined novel editing sites in the nucleoprotein (NP) and L open reading frames, increasing the potential coding capacity of these viral genes with as-yet-unknown functions ^20^.

### b.Marburg virus assembly

The MARV RNA genome is in close association with the viral nucleocapsid proteins, including NP (enwraps viral RNA), the RNA-dependent RNA polymerase complex consisting of L (enzymatic moiety of the polymerase complex), and viral protein 35 (VP35) (polymerase cofactor), VP30 (function not clear; possibly a transcription regulator), and VP24 (involved in nucleocapsid formation and maturation). Viral genome replication and transcription occur in the cytoplasm of the infected cells in viral inclusions which are highly ordered aggregations of nucleocapsids ^22^. Live-cell imaging has shed some light on the trafficking of mature MARV nucleocapsids from viral inclusions to the sites of budding and helped to identify viral and cellular proteins involved in this process. Mature MARV nucleocapsids are transported along actin filaments from the viral inclusions to the plasma membrane, where they recruit the viral matrix protein VP40. Only nucleocapsids that are associated with VP40 are transported into filopodia ^23^. These long cellular protrusions are the main budding sites of MARV particles. MARV NP contains a late domain motif (PSAP) that recruits tumor susceptibility gene 101 (Tsg101), a component of the vesicular transport system ESCRT-I (endosomal sorting complexes required for transport I), to the viral inclusions. NP–Tsg101 interaction is required for the actin-dependent transport of MARV nucleocapsids into the filopodia ^24^. The MARV glycoprotein (GP) is also recruited to VP40-enriched membranes by a tubulin-dependent process ^25^.

### c. Structure–function of Marburg virus proteins

Structural analyses have provided a deeper insight into the interactions and functions of almost all seven MARV proteins ^26^. Comparison of the MARV proteins with their ebolavirus homologs has shown a varying degree of structural resemblance that often correlates with functional similarity ^27– 36^. Intriguingly, despite considerable structural conservation, some of the MARV proteins are functionally different from their ebolavirus homologs, with VP24 being a prime example of this.

***Viral protein 24.*** VP24 is required for nucleocapsid formation and assembly for both viruses, but only ebolavirus VP24 blocks type I interferon (IFN) signaling (reviewed in 37, 38). Crystal structure analysis revealed that the ebolavirus and MARV VP24 cores are structurally similar, supporting their common function in nucleocapsid formation and assembly. One difference between MARV and ebolavirus VP24 proteins is an extended β-sheet that is formed by MARV VP24 amino acid residues 201 to 217 ^35^. Although this structural difference does not explain why MARV VP24 is not immunosuppressive ^35^, it is connected to a novel function of MARV VP24. The extended β-shelf contains the binding domain for the cellular adaptor protein Kelch-like ECH-associated protein 1 (Keap1). Keap1 is a central player in the oxidative stress response pathway and a suppressor of the antioxidant response transcription factor nuclear factor (erythroid-derived 2)-like 2 (Nrf2). MARV VP24 binds to Keap1 via the “K loop” that comprises amino acids 202 to 209 ^39, 40^. This leads to the nuclear accumulation of Nrf2 and consequently to the activation of an antioxidative and cytoprotective response in MARV-infected cells ^39, 41^. In contrast, ebolavirus VP24 proteins, which do not have the K loop, are not able to induce a cytoprotective response ^39, 41^. Keap1 is also a regulator of the nuclear factor kappa-light-chain-enhancer of activated B cells (NF-κB) pathway. It targets IκB kinase β (IKKβ) for degradation which disables the cell to respond to NF-κB–activating stimuli. MARV VP24 prevents Keap1 from binding to IKKβ, allowing the cell to respond to NF-κB–activating stimuli ^42^.

***Viral protein 30.*** Another example for functional difference despite structural conservation is VP30. Although EBOV VP30 has been shown to act as a transcription activator and regulator of viral RNA synthesis ^16, 32^, the role of MARV VP30 is less well understood. Despite the high degree of structural homology between MARV and EBOV VP30, EBOV VP30 enhances transcriptional activity in a minigenome system at least 50-fold and is essential for detectable reporter gene activity, whereas MARV VP30 increases reporter gene activity only moderately (about twofold) in the MARV minigenome system ^43– 45^. However, MARV VP30 is essential for viral replication ^46, 47^. Crystal structure analysis confirmed the conformational similarity of EBOV and MARV VP30 proteins. Both EBOV and MARV VP30 C-terminal domains bind a peptide in the respective NP C-terminal region. The EBOV and MARV NP–VP30 complexes are remarkably similar, although slight differences might account for the lower binding affinity observed for the MARV NP–VP30 complex (K _D_ of 14 µM compared with K _D_ of 5.7 µM for EBOV complex). NP–VP30 complex formation was shown to be essential for EBOV minigenome activity ^32^. Whether the observed differences in VP30–NP binding affinity play a role in the lower efficiency of MARV VP30 to enhance transcription remains to be determined.

Both EBOV and MARV VP30 proteins are phosphorylated, and phosphorylation of EBOV VP30 blocks its transcriptional activity ^48– 52^. A recent study showed that phosphorylation of MARV VP30, like that of EBOV, interferes with its enhancing function in the minigenome system ^45^.

***Viral protein 35.*** MARV VP35 plays an important role in various steps of the viral replication cycle. It is a component of the viral RNA-dependent RNA polymerase, required for nucleocapsid formation, and suppresses antiviral host responses, including type I IFN production, protein kinase R (PKR) activation, and dendritic cell maturation ^53– 56^. Crystal structure analysis provided deeper insights into the various functions of MARV VP35. Similar to EBOV VP35 ^57, 58^, MARV VP35 chaperones NP by inhibiting NP oligomerization and RNA binding. This facilitates the release of NP-bound viral RNA and gives the viral polymerase access to the RNA template. It also prevents unspecific NP-RNA aggregation ^33, 34^. In regard to its function as a suppressor of type I IFN production, MARV VP35 seems to be less efficient than its EBOV homolog ^59– 61^. This difference might be due to differences in the binding modes and affinities of EBOV and MARV VP35 proteins to double-stranded RNA, potentially impacting the inhibition of downstream antiviral pathways ^59, 60, 62^. Interestingly, compared with MARV VP35, RAVV VP35 seems to be more efficient in suppressing type I IFN production ^61^.

***Viral protein 40.*** MARV VP40 is a multifunctional protein that mediates virus egress, regulates viral transcription and replication, and counteracts the innate immune response by blocking signal transducer and activator of transcription (STAT)1 and STAT2 signaling ^63^. Recent research on VP40 has focused on the mechanisms underlying VP40–membrane interaction and budding. Crystal structure analysis has shown that MARV VP40 in solution forms a dimer with an N-terminal dimer interface. The C-terminal domains of the VP40 dimer, which form a flat cationic surface that interacts with anionic phospholipids at the inner leaflet of the plasma membrane ^29, 36, 64^, are required for budding but not for the immunosuppressive function of VP40 ^29^. Hydrogen–deuterium exchange mass spectrometry has suggested that this interaction facilitates VP40 assembly and oligomerization ^65^.

VP40 hijacks cellular vesicular transport systems to bud from the infected cells, including the coat protein complex II (COPII) vesicular transport system and the ESCRT system ^37^. A screen to find additional cellular proteins that bind to the late domain motif (PPxY) within EBOV VP40 identified the co-chaperone protein BCL2-associated athanogene 3 (BAG3). In contrast to other late domain binding proteins that promote filovirus budding, BAG3 inhibits EBOV and MARV VP40-mediated budding and therefore could be part of a cellular antiviral defense system ^66^.

It has been shown previously that VP40 is a species-specific virulence factor and that changes in VP40 are required for marburgvirus adaptation to rodent hosts (reviewed in 67; an overview of the various MARV and RAVV isolates is provided in 7). Mouse adaptation of two marburgvirus isolates—RAVV and MARV Ci67—led to several mutations in the VP40 gene ^68, 69^. Although the wild-type versions of RAVV and MARV Ci67 VP40 antagonized the type I IFN response inefficiently in mouse cells, the mouse-adapted VP40 mutants retained this function in both human and mouse cells ^70, 71^. Intriguingly, the underlying amino acid changes required to facilitate type I IFN antagonism in mouse cells differed in the mouse-adapted RAVV and MARV Ci67 VP40 proteins ^70, 71^. Other functional changes that correlated with mouse adaption affected the ability of VP40 to mediate budding. Whereas wild-type RAVV VP40 efficiently mediated budding from both human and mouse cells, mouse-adapted RAVV VP40 was restricted in this function by tetherin in human cells ^72^. Mutations in VP40 induced by guinea pig adaptation of MARV Musoke resulted in increased budding and a gain of viral fitness in guinea pig cells while not altering the type I IFN antagonizing function of VP40 in human or guinea pig cells ^73^. This shows that VP40 mutations which occur during rodent adaptation not only enhance type I IFN antagonism in these hosts but also promote VP40-mediated budding. MARV adaptation to guinea pigs resulted in mutations in both VP40 and the viral polymerase (L). Minigenome data suggest that there is a synergistic effect of these mutations in guinea pig cells, leading to enhanced replication activity ^74^. Because of the role of VP40 as a virulence factor, the inhibition of type I IFN signaling by VP40 represents an intriguing potential target for the development of anti-MARV therapeutics. Unfortunately, this is currently limited by our poor understanding of the exact molecular mechanisms underlying this inhibition.

***Glycoprotein.*** Given its exposure on the surface of viral particles, which makes it the perfect target for antibody recognition, and its crucial role in attachment and fusion, MARV GP has been extensively studied in the last 6 years. This includes the identification and characterization of host factors involved in attachment and entry ^75– 77^, the role of GP _2_ in membrane fusion and tetherin antagonism ^30, 31, 78, 79^, and the effects of steric shielding of host surface proteins, including major histocompatibility complex I, integrin β1, and Fas, by MARV GP ^80, 81^. GP shielding of surface-expressed Fas interfered with the induction of Fas-mediated apoptosis and could help to protect MARV-infected cells from premature cell death ^81^. Interestingly, steric shielding of host proteins was more pronounced for MARV Angola GP than for MARV Musoke GP, indicating that it might play a role in virus-specific pathogenicity ^80^ (see section 3b). Much progress has also been achieved in the structural analysis of GP and its interaction with protective antibodies, which are promising candidates for post-exposure treatment ^82, 83^. An excellent overview by King *et al*. on the structural features of protective epitopes on filovirus GPs highlights the striking differences between the neutralizing epitopes on EBOV and MARV GPs ^84^. To date, only two MARV GP epitopes that are targeted by protective antibodies have been identified: the receptor binding site and the wing domain that is located within the MARV GP _2_ subunit and is not present in ebolavirus GPs. The MARV receptor binding site seems to be easily accessible to neutralizing antibodies whether GP is cleaved or not, whereas the respective region on ebolavirus GPs is shielded by a glycan cap and is exposed only after GP cleavage ^85, 86^. The receptor binding site antibodies have shown promising potential for use as therapeutics, as they are protective when administered up to 5 days post-exposure in NHPs ^83^. Testing of antibodies directed against the wing domain have been less encouraging: protection of mice is observed when given 1 hour after infection ^87^, highlighting a large disparity in therapeutic potential between antibodies directed against these two regions of GP. Owing to differences in structure and identified protective epitopes, the search for a pan-filovirus antibody has remained unfruitful so far, although pan-ebolavirus protective antibodies have been identified ^88– 95^.

## 2. Host response to Marburg virus infection

Recent studies on the host response to MARV infection have focused on identifying signatures of innate and adaptive immunity as well as correlates of disease severity and outcome in both human MVD survivors and MARV-infected NHPs. Although both EBOV and MARV infections are lethal in cynomolgus macaques, transcriptional analysis revealed unique immune signatures associated with each virus and suggested a more pronounced immune dysregulation in MARV infection ^96^. The MARV-specific gene expression profile included the upregulation of complement system genes, genes involved in neutrophil and monocyte recruitment, and innate immune signaling genes ^96^. Distinct immune responses, which are predictive of clinical outcomes, have been detected in both human survivors and macaques infected with MARV: although lethal infection of rhesus macaques with MARV Angola was linked to T-helper cell type 2 (Th2)-skewed responses ^97^, human survivors of MARV infection exhibited Th1-skewed CD4 ^+^ T-cell responses ^98^. The ability to identify these predictive responses in patients could result in more effective patient triage and administration of targeted therapies.

Another major focus of MVD research has been the pursuit of earlier diagnostics, and particular attention has been on the early identification of unique signatures of marburgvirus infection. MARV infection of NHPs led to the activation of peripheral blood mononuclear cells, as reflected by the induction of the type I IFN response in rhesus macaques ^99^ and increased expression of heat shock proteins in cynomolgus macaques ^97, 100^. Upregulation of type I IFN-stimulated genes was detected as early as 1 day post-infection in MARV-infected rhesus macaques, prior to the onset of symptoms ^97^. These data indicate that new diagnostic tools could be developed to detect these unique signatures of marburgvirus infections prior to the onset of viremia or symptoms, allowing earlier detection and treatment of MVD.

A major unanswered question in the filovirus field is how long human survivors remain protected against subsequent exposure to autologous virus. Although long-lived antibody responses have been found in human survivors of Sudan and Bundibugyo viruses (both ebolaviruses) as well as MARV ^101^, MARV neutralizing antibody responses were lower and showed a faster decline in contrast to the prolonged presence of neutralizing antibodies after Sudan virus infection ^98, 102^. Low titers of neutralizing antibodies were also observed in cynomolgus macaques after vaccination against MARV. Nevertheless, animals were protected against lethal MARV challenge for over 1 year ^103^. These findings emphasize the need for a better understanding of the differences in host response to diverse filoviruses and to determine long-term effects of vaccine candidates.

## 3. Marburg virus prevalence and pathology in bats

Another area of progress in the marburgvirus field is focused on the natural reservoir of these viruses. Prior work showed that the common Egyptian fruit bat ( *Rousettus aegyptiacus*) is a reservoir for marburgviruses, having demonstrated positive serology, detection of viral RNA, and the ability to culture infectious virus from the bats, a feat still unparalleled in the ebolavirus field. Since our previous review of the marburgvirus literature ^7^, major advances have been made in our understanding of the ecology of marburgviruses in Egyptian fruit bats, including the outcome of experimental infection of these bats with MARV.

### a. Ecology of marburgviruses in bats

Expanding upon the handful of reports prior to 2012, more recent studies have investigated the prevalence of marburgviruses in various bat species. These reports primarily investigated the prevalence of Egyptian fruit bat exposure to MARV and RAVV as determined by serology and, to a lesser extent, quantitative real-time polymerase chain reaction (RT-PCR), finding varying degrees of prevalence in different geographic populations. The finding of higher rates of Egyptian fruit bats with detectable marburgvirus RNA that repopulated Kitaka mine ^104^ versus the population that was present prior to extermination ^105^ led the authors to propose that the new founding population may have been susceptible to MARV infection and, following multiple introductions of diverse marburgviruses, led to the high rates of RT-PCR–positive bats. This finding should serve as a warning for possible future attempts at controlling MARV outbreaks by trying to eradicate local bat colonies; such an approach may backfire.

Three separate studies analyzing MARV seropositivity and RNA in Egyptian fruit bats in Uganda, South Africa, and Zambia all detected cyclical temporal patterns: the lowest prevalence was observed during the birthing season and prevalence increased thereafter, particularly among juvenile bats ^106– 108^. Intriguingly, 83% of previous marburgvirus outbreaks in the human population coincided with these peaks in viral abundance in Egyptian fruit bats ^106^, further implying the importance of Egyptian fruit bats as the prime source for MARV spillover to humans. The fact that similar data were obtained in diverse bat ** populations that differ drastically in their geographic distribution and mating patterns (once versus twice per year) strengthens the possibility that marburgvirus prevalence, at some level, may be linked to behavioral patterns of these bats.

Because MARV infection appears to be quite mild in Egyptian fruit bats (see section 2b) and prevalence is fairly low, some have speculated either that *R. aegyptiacus* is not the reservoir species or that it is unlikely to be the only species required for the enzootic maintenance of marburgviruses. To date, however, investigations of marburgvirus seroprevalence in other bat species ^105, 109^ and marburgvirus presence in *R. aegyptiacus*–associated ticks ( *Ornithodoros faini* ^110^) have been unfruitful in finding other likely reservoir species.

### b. Experimental infection of Egyptian fruit bats

Critical advances in our understanding of MARV infection of Egyptian fruit bats have been facilitated by the establishment of captive, breeding colonies of Egyptian fruit bats at the Center for Emerging and Zoonotic Diseases in Sandringham, South Africa, and at the Centers for Disease Control and Prevention in Atlanta, Georgia, USA. Studies reporting primary MARV infection of Egyptian fruit bats from these two colonies have yielded strikingly similar results; although MARV is able to replicate in bats, viremia remains low, there is no resulting sickness or pathology in the bats, and the bats are able to quickly clear the infection ^111– 114^. Whereas both groups observed MARV RNA in oral and rectal swabs of infected Egyptian fruit bats ^112, 114^, only one of the groups was able to culture live virus from oral and rectal swabs from the infected bats ^112^.

An important aspect in considering the ecology of MARV in *R. aegyptiacus* populations is whether prior infection with MARV prevents subsequent reinfection. Two separate challenge studies clearly showed that Egyptian fruit bats previously infected with MARV were resistant to challenge by a homologous MARV isolate ^115, 116^.

### c. Transmission of Marburg virus by Egyptian fruit bats

Recent studies investigating the ability of MARV-infected Egyptian fruit bats to spread the virus to uninfected, co-housed animals have resulted in more divergent results. Whereas Paweska *et al*. found no evidence of transmission despite robust infection of the injected bats ^114^, Schuh *et al*. did ^117^. MARV was detected in blood and oral samples from the bats that were infected by horizontal transmission, providing a possible mechanism of viral transmission (saliva transmission). The different outcomes could be due to the different virus isolates used in these studies or differences in the genetic background of the bats themselves.

### d. Molecular biology of Marburg virus infection in bat cells

In addition to advances in studying infections in bats, much recent study has focused on cellular responses in bat cells to MARV infection. Multiple transcriptomic analyses of MARV infection of *R. aegyptiacus* cell lines have come to similar conclusions. Infection of the cell lines R06E (derived from fetal body cells) ^118^ and R0Ni/7.1 (adult kidney) ^119^ appears to follow patterns similar to those of transcriptional responses in human cells, although viral replication was not as robust in the bat cells as in Huh7 and HepG2 human cells ^120– 122^. Whereas two of these reports found either no induction or minimal induction of type I, II, or III IFNs or IFN-stimulated genes in bat cells infected with MARV ^120, 121^, one of these reports found low to modest upregulation of these genes ^122^. This may be due to differences in experimental design or differences in the MARV isolates used in the studies. Interestingly, one of these reports found that MARV upregulated some unannotated antiviral paralogs ^121^.

Despite a relative dearth of bat-specific antibodies, some non-transcriptomic analyses have begun analyzing molecular mechanisms of MARV infection in bat cell lines. Although EBOV VP35 had previously been shown to inhibit PKR in human cells, a recent report describes the ability of MARV VP35 to inhibit PKR activation in a cell type–specific manner, with VP35 being unable to inhibit bat PKR in the RoNi/7.1 cell line ^55^. An analysis of fibroblast cell lines from four different bat species revealed that species-specific differences in Niemann–Pick C1 (NPC1) accounted for differences in EBOV GP-mediated, but not MARV GP-mediated, infectivity ^123^.

## 4. Advances in animal modeling of marburgvirus infections

Historically, individual studies analyzing marburgviruses have been performed using single isolates of MARV or RAVV, and comparative studies have been sparse ^124^. However, fueled by discussions about the impact of viral sequence variations on pathogenic potential that arose during the West African EBOV outbreak ^125– 128^ and by the advent of high-throughput sequencing technologies, marburgvirus isolate variations are now more acknowledged, and marburgvirus diversity has become a research topic. Well-established and newly developed animal models are useful tools to conduct these comparative studies. A list of marburgvirus variants commonly used in animal experiments during the last 6 years is provided in Table 1.

**Table 1.** Experimentally used marburgviruses.

Year	Country of origin	Country of isolation	Isolate	Reference
1967	Presumably Uganda	Germany	MARV Ci67 (Marburg virus/H.sapiens-tc/ GER/1967/Hesse-Cieplik)	Lofts *et al*. (2011) ^68^, Coffin *et al*. (2018) ^139^
			MARV Voege (Marburg virus/H.sapiens-tc/ GER/1967/Hesse-Voege)	Atkins *et al*. (2018) ^132^
			MARV Pop (Marburg virus/H.sapiens-tc/ GER/1967/Hesse-Popp)	Smither *et al*. (2013) ^134^
1975	Rhodesia (now Zimbabwe)	South Africa	MARV Ozolins (Marburg virus/H.sapiens-tc/ ZAF/1975/Sinoia-Ozolins)	Nicholas *et al*. (2018) ^140^
			MARV Hogan (Marburg virus/H.sapiens-tc/ ZAF/1975/Sinoia-Hogan)	Paweska *et al*. (2012) ^111^
1980	Kenya	Kenya	MARV Musoke (Marburg virus/H.sapiens-tc/ KEN/1980/Mt. Elgon-Musoke)	Atkins *et al*. (2018) ^132^, Blair *et al*. (2018) ^141^, Nicholas *et al*. (2018) ^140^, Daddario-DiCaprio *et al*. (2006) ^142^, Coffin *et al*. (2018) ^139^, Daddario-DiCaprio *et al*. (2006) ^143^, Mire *et al*. (2014) ^103^
1987	Kenya	Kenya	RAVV (Ravn virus/H.sapiens-tc/KEN/1987/ Kitum Cave-810040)	Atkins *et al*. (2018) ^132^, Lofts *et al*. (2011) ^68^, Cross *et al*. (2015) ^144^, Nicholas *et al*. (2018) ^140^, Daddario-DiCaprio *et al*. (2006) ^142^, Thi *et al*. (2017) ^145^, Mire *et al*. (2017) ^83^
1998–2000	COD	COD	MARV SPU 148/99/1	Paweska *et al*. (2015) ^114^, Storm *et al*. (2018) ^116^
2004–2005	Angola	Angola	MARV-Angola-AGO-2005	Qiu *et al*. (2014) ^146^, Wong *et al*. (2018) ^147^
			MARV-Angola-AGO-2005-368	Lavender *et al*. (2018) ^133^, Wong *et al*. (2018) ^136^, Marzi *et al*. (2016) ^148^, Fernando *et al*. (2015) ^149^, Nicholas *et al*. (2018) ^140^, Marzi *et al*. (2018) ^96^
			MARV Angola (Marburg virus/H.sapiens-tc/ AGO/2005/Angola-1379c)	Atkins *et al*. (2018) ^132^, Johnston *et al*. (2015) ^150^, Lin *et al*. (2015) ^151^, Alfson *et al*. (2018) ^152^, Cross *et al*. (2015) ^144^, Blair *et al*. (2018) ^141^, Cooper *et al*. (2018) ^153^, Woolsey *et al*. (2018) ^154^, Thi *et al*. (2017) ^145^, Mire *et al*. (2017) ^83^
			MARV-Angola-1381	Ewers *et al*. (2016) ^155^
2007	Uganda	Uganda	MARV-371-Bat2007 (Marburg virus/ R.aegyptiacus-tc/UGA/2007/371Bat-811277)	Amman *et al*. (2015) ^112^, Jones *et al*. (2015) ^113^, Schuh *et al*. (2017) ^115, 117^

This table shows Marburg virus (MARV) and Ravn virus (RAVV) variants that have been used in experimental animal infections highlighted in this review. “tc” in the virus name indicates tissue culture passaging. This information is not available for all listed viruses, but it is likely that all isolates were passaged more than once. COD, Democratic Republic of the Congo.

### a. Newly developed animal models to study Marburg virus and Ravn virus infection

Since our previous review in 2012, a number of new animal models of MVD have been developed. Detailed comparisons of these and previously established MVD animal models have recently been published ^129– 131^. Instead of repeating the content of these comprehensive reviews, we will briefly introduce these animal models and then focus on their use in resolving isolate-specific differences in virulence and pathogenicity as well as their use in modeling post-recovery sequelae.

Recently developed animal models that allow the study of lethal MVD without virus adaptation include STAT2 knockout hamsters ^132^, humanized mice ^133^, and marmosets ^134^. Infection of humanized mice with MARV, compared with infection with EBOV, was associated with lower overall weight loss, whereas the viral titers were similar ^133^. Surprisingly, ferrets infected with MARV or RAVV, in contrast to those infected with EBOV, did not develop symptoms of disease, regardless of dose, route of infection, and virus isolate ^135– 138^. Immunocompetent small-animal infection models for MARV and RAVV, including mice, hamsters, and guinea pigs, require virus adaptation. Recent virus-adapted systems include a hamster model for MARV Angola that recapitulates the disease observed in humans and NHPs ^148^ and MARV Angola mouse models ^146, 156^. Similar to what was observed before for mouse-adapted MARV isolates Ci67 and RAVV ^67^, deep sequencing of the MARV Angola genome during mouse adaptation revealed, among other mutations, adaptive changes in the VP40 open reading frame ^68, 156^, emphasizing the importance of VP40 as a species-specific virulence factor (see “Viral protein 40” section above).

### b. Differential virulence of Marburg virus variants

MARV Musoke was isolated in 1980 in Kenya from a physician who contracted the disease while attending a patient with MVD. Whereas the index case succumbed to the infection, the secondary case survived. There is only a single virus isolate from this MVD episode, and it was isolated from the surviving patient ^157^. The MARV Angola variant comes from the 2005 outbreak of MVD in Angola with at least 252 cases, including 227 fatalities. Multiple virus isolates exist from this outbreak ^157^. It is important to note that even individual isolates may vary from lab to lab, as they have typically been passaged in cell culture multiple times, disseminated to other laboratories, and possibly passaged further, leading to a diversity of virus stocks with different histories of propagation and thus potentially divergent genetic and phenotypic identities ^152^. Passaging of viruses in cell culture is known to lead to the accumulation of defective interfering viral particles and higher particle/plaque-forming unit ratio ^99^. This was also shown for MARV Angola and EBOV; virus passaged multiple times in cell culture was found to be associated with increased survival and delayed disease progression in NHPs ^152, 158^. Given the much longer history of the stocks derived from the MARV Musoke isolate, it is conceivable that MARV Musoke stocks have accumulated defective particles over time and that the compositions of the MARV Musoke and MARV Angola virus stocks differ, which should be taken into account when interpreting differences in pathogenicity.

Comparative studies in small-animal models that require virus adaptation revealed a range in pathogenicity, with MARV Angola being more pathogenic than other marburgvirus variants, including RAVV ^144, 146, 147^. Different results were obtained using the STAT2-deficient hamster infection model that does not require virus adaptation. In this infection model, fast disease progression and death were observed with MARV Musoke and Voege, a delayed but equally lethal infection was observed with MARV Angola, and RAVV resulted in a symptomatic but non-lethal infection ^132^.

The pathogenicity of different marburgvirus variants was also studied in NHP models which recapitulate the disease in humans most faithfully. Due to advances in telemetry tracking of clinical data, disease symptoms now can be more easily monitored in this infection model ^155^. Fatal MARV infection is associated with increased lymphocyte and hepatocyte apoptosis in both rhesus and cynomolgus macaques ^140^. In the rhesus and cynomolgus macaque models, as in the small-animal models using adapted marburgviruses, MARV Angola showed higher virulence compared with other marburgvirus variants, including MARV Musoke, Ci67, Ozolin, and RAVV ^140– 143, 149, 154, 159^. The increased virulence of MARV Angola could be explained, at least partially, by an enhanced replication efficiency of this virus, as shown in cell culture experiments ^160^. Conflicting results have been reported for RAVV infections of NHPs. Although RAVV infection of rhesus macaques was 100% lethal in two studies ^83, 145^, complete survival was noted in a separate study ^140^. Overall, these studies highlight the need for further comparative analysis of different marburgvirus variants in different animal models.

### c. Persistent Marburg virus infection in non-human primate models

Although persistent MARV infection and long-lasting health issues after recovery have been noted for a number of survivors of MVD, including those from the initial outbreak in 1967 ^3^, this issue became a research topic only after the devastating EVD outbreak in West Africa. Recent studies using rhesus macaques have shown that immune-privileged sites, including the eyes, female and male genital tracts, and mammary glands, are infected during acute MARV infection ^153^. Similar to human survivors of EBOV and MVD ^161– 163^, some cynomolgus macaque survivors (including those that had been treated in antiviral drug testing) developed persistent MARV infection in the eyes and testes ^139^. These results indicate that NHP survivors of experimental MARV infection could serve as useful models to study sequelae of MVD. It remains to be determined whether persistent infection also occurs in other animal models of filovirus infection.

## Abbreviations

BAG3, BCL2-associated athanogene 3; EBOV, Ebola virus; ESCRT, endosomal sorting complexes required for transport; EVD, Ebola virus disease; GP, glycoprotein; IFN, interferon; IKKβ, IκB kinase β; Keap1, kelch-like ECH-associated protein 1; MARV, Marburg virus; MVD, Marburg virus disease; NF-κB, nuclear factor kappa-light-chain-enhancer of activated B cells; NHP, non-human primate; NP, nucleoprotein; Nrf2, nuclear factor (erythroid-derived 2)-like 2; PKR, protein kinase R; RAVV, Ravn virus; RT-PCR, real-time polymerase chain reaction; STAT, signal transducer and activator of transcription; Tsg101, tumor susceptibility gene 101; VP, viral protein
